# Opportunities and Challenges in the Delivery of mRNA-Based Vaccines

**DOI:** 10.3390/pharmaceutics12020102

**Published:** 2020-01-28

**Authors:** Abishek Wadhwa, Anas Aljabbari, Abhijeet Lokras, Camilla Foged, Aneesh Thakur

**Affiliations:** Department of Pharmacy, Faculty of Health and Medical Sciences, University of Copenhagen, Universitetsparken 2, DK-2100 Copenhagen Ø, Denmark

**Keywords:** mRNA, vaccines, therapeutic, prophylactic, drug delivery systems, lipids, polymers, nanoparticles, nanomedicine

## Abstract

In the past few years, there has been increasing focus on the use of messenger RNA (mRNA) as a new therapeutic modality. Current clinical efforts encompassing mRNA-based drugs are directed toward infectious disease vaccines, cancer immunotherapies, therapeutic protein replacement therapies, and treatment of genetic diseases. However, challenges that impede the successful translation of these molecules into drugs are that (i) mRNA is a very large molecule, (ii) it is intrinsically unstable and prone to degradation by nucleases, and (iii) it activates the immune system. Although some of these challenges have been partially solved by means of chemical modification of the mRNA, intracellular delivery of mRNA still represents a major hurdle. The clinical translation of mRNA-based therapeutics requires delivery technologies that can ensure stabilization of mRNA under physiological conditions. Here, we (i) review opportunities and challenges in the delivery of mRNA-based therapeutics with a focus on non-viral delivery systems, (ii) present the clinical status of mRNA vaccines, and (iii) highlight perspectives on the future of this promising new type of medicine.

## 1. Introduction

Vaccination has had a tremendous impact on global health and the quality of human life by reducing the mortality and morbidity caused by infectious diseases. The development of vaccines is predicated on the classical 3I’s paradigm of “isolating, inactivating and injecting” the causative microorganism, coined by Louis Pasteur [1]. Vaccines can be prophylactic or therapeutic and can broadly be classified as live attenuated vaccines (weakened microorganisms), inactivated vaccines (killed microorganisms), subunit vaccines (purified antigens), or toxoid vaccines (inactivated bacterial toxins). As opposed to the conventional concept of injecting live-attenuated or inactivated pathogens, modern vaccine approaches, i.e., subunit vaccines, focus on exhibiting efficacy similar to conventional vaccines while obviating the safety risks associated with whole-cell vaccines. However, subunit antigens often display lower immunogenicity, which can be rectified by employing delivery systems and/or immunopotentiating compounds as adjuvants to boost immunogenicity. The modern genome-based rational vaccine design offers tremendous potential over conventional whole-organism-based vaccine approaches. 

Nucleic acid-based vaccines, i.e., DNA (as plasmids) and RNA (as messenger RNA (mRNA)) vaccines, pave the way for safe and efficacious biologics to mimic inoculation with live organism-based vaccines, particularly for stimulation of cell-mediated immunity [2]. This technology exhibits promising potential for the development of novel vaccines against a wide variety of indications and diseases, extending from prophylactics to therapeutics for infectious diseases, cancer, autoimmune diseases, and hypersensitivities. While nucleic-acid-based vaccines demonstrate significant advantages over traditional vaccines [3] in terms of safety, efficacy, induction of both B- and T-cell responses and specificity, it is noteworthy to mention that mRNA vaccines have advantages when compared to vaccines based on other types of nucleic acids. A technical challenge associated with DNA vaccines is to ensure delivery into the cell nucleus, where antigen transcription takes place prior to nuclear export and translation into protein in the cytoplasm. In addition, DNA vaccines carry a potential risk of integration into the host genome, which may result in insertional mutagenesis. In contrast, mRNA vaccines are only targeted for cytoplasmic delivery, circumventing the risk of genomic integration [4]. The relatively short half-life results in transient and more controlled expression of the encoded antigen. Moreover, mRNA can be produced in a cell-free environment by in vitro transcription (IVT), thereby eschewing the use of microbes or cultured cells for production, and avoiding the associated quality and safety issues in the production. This permits simple downstream purification and rapid and cost-effective manufacturing [5]. However, mRNA is often promulgated on the grounds of the popular opinion that when using mRNA, unlike DNA, the stringent gene-therapy regulations are bypassed because mRNA does not integrate into the host genome. However, in reality, this only holds true in the US since in Europe, any active pharmaceutical ingredient, which contains or consists of a recombinant nucleic acid, used in or administered to human beings, falls under the scope of the regulation for advanced therapy medicinal products [6]. Therefore, mRNA-based therapeutics are categorized as gene therapy. The burgeoning field of mRNA vaccines is very exciting [3,7] and considerable amounts of relevant preclinical data have been generated, and several clinical trials have been initiated during the last decade. This gives rise to the vision of translating the mRNA vaccines into human application for prophylaxis and therapy. In this review, we discuss the current trends in mRNA vaccine approaches and various strategies and systems for delivering mRNA vaccines. 

## 2. mRNA as Vaccines

The central dogma of molecular biology states that DNA is transcribed into mRNA, which is subsequently translated into protein [8]. The flow of genetic information in time and space is orchestrated by complex regulatory mechanisms. Gene therapy represents the introduction of genetic material into an individual’s cells and biological tissues. Techniques like insertion, alteration, or removal of genes are employed for correcting defective genes responsible for disease development, which then cures a disease or ameliorates the clinical status of a patient [9]. Several vectors have been utilized for gene therapy and they are generally classified as non-viral and viral vectors. Non-viral vectors possess several advantages compared to the viral vectors, including low host immunogenicity and potential for scale-up [10]. However, the success of non-viral gene therapy has been very limited in the past, primarily due to the barriers existing for plasmid DNA (pDNA) delivery, e.g., the necessity to cross the nuclear membrane before translation, the presence of antibiotic resistance genes in pDNA, and most importantly, the difficulty in controlling and regulating long-term expression. The lack of control of long-term expression of pDNA poses a huge disadvantage in terms of the duration of treatment and possible side effects, which is in contrast to conventional drugs, where the treatment can be stopped instantaneously. These disadvantages of pDNA can possibly be overcome by using mRNA [11]. The mRNA carries genetic information from the DNA in the nucleus to the cytosol, where it is used by the ribosomes as a template for protein synthesis. As opposed to pDNA, the mRNA is efficacious in both mitotic and non-mitotic cells because mRNA exerts its function in the cytoplasm, hence its function is not dependent on active cell division. Furthermore, unlike pDNA or viral vectors, mRNA does not contain additional foreign genes, which makes mRNA a safer vector. The challenge of long-term expression posed by pDNA can also be overcome by using mRNA, since mRNA mediates a rapid, transient expression of the encoded protein and the duration of the production is well-defined (usually a few days or weeks, depending on the specific mRNA platform). This makes mRNA expression easier to control than the gene expression from pDNA and viral vectors [11]. In addition, the manufacturing of mRNA is cell-free, which strongly reduces the chance of mRNA contamination with bacterial components. This makes it easier to produce mRNA than pDNA under good manufacturing practice conditions [12]. Finally, vector-induced immunogenicity can be avoided for mRNA therapeutics, unlike for viral vectors or virus-like particles, which may elicit a specific immune response against the exposed viral proteins [13]. Specific therapeutic applications of mRNA, which are currently being explored include (i) vaccination against cancer and infectious diseases, (ii) protein-replacement therapy, and (iii) gene editing. Table 1 summarizes examples of ongoing clinical trials of mRNA-based therapeutic and prophylactic vaccine candidates.

Two classes of mRNAs, i.e., non-replicating and self-amplifying mRNA, are commonly used as vaccine vectors. Non-replicating mRNA encodes only the protein antigen(s) of interest, while self-amplifying mRNA also encodes proteins enabling RNA replication [14]. Vaccines based on self-amplifying mRNA encode the RNA genome of a single-stranded RNA virus, e.g., an alphavirus, a flavivirus [15], or a picornavirus [7]. They are engineered to increase the duration and level of expression, as well as the subsequent immune response induced by the encoded antigen(s). They efficiently amplify the production of sub-genomic mRNA encoding antigen(s) of interest subsequent to a single round of replication. While both self-amplifying mRNA and non-replicating mRNA find application in prophylactic vaccines for infectious diseases, non-replicating mRNA is used for cancer vaccines.

## 3. Fundamental Pharmacology of mRNA Vaccines 

In vitro transcribed (IVT) mRNA is employed therapeutically as it mimics fully mature native mRNA present in the eukaryotic cytosol [16]. This may be achieved either by ex vivo transfection of cells with mRNA that are then adoptively transferred or by direct in vivo delivery of the IVT mRNA to the cytosol [17]. These approaches are explored for genome engineering, genetic reprogramming, adoptive T cell and dendritic cell (DC) based cancer and infectious disease immunotherapies, tolerization regimens to treat allergies, and protein replacement therapies. Both ex vivo transfection and direct in vivo transfection enable the target cells to synthesize the encoded protein(s) in situ, where mRNA is used as a template and the protein(s) represents the active product. The open reading frame (ORF) of mature mRNA encoding the protein(s) of interest (the active product) marked by start and stop codons, respectively, is flanked by untranslated regions (UTRs), and ideally consists of a 5’ cap and a poly(A) tail [3].

**Figure 1:**
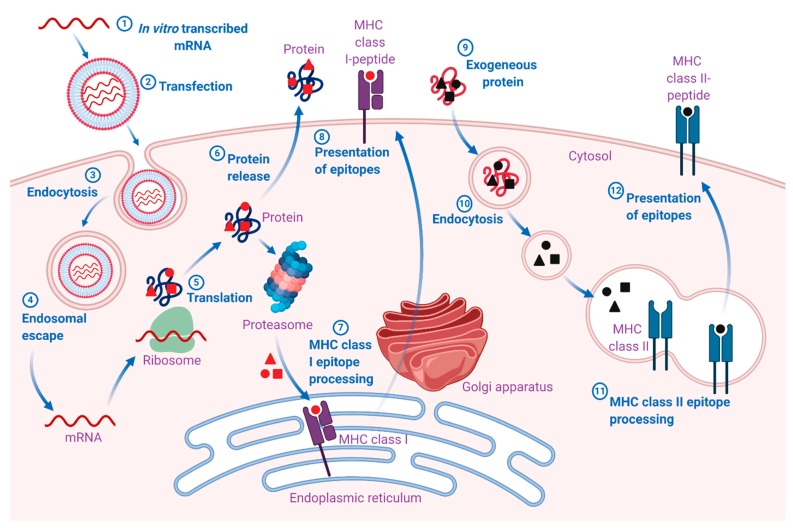
Mechanism of action of mRNA vaccines. 1. The mRNA is in vitro transcribed (IVT) from a DNA template in a cell-free system. 2. IVT mRNA is subsequently transfected into dendritic cells (DCs) via (3) endocytosis. 4. Entrapped mRNA undergoes endosomal escape and is released into the cytosol. 5. Using the translational machinery of host cells (ribosomes), the mRNA is translated into antigenic proteins. The translated antigenic protein undergoes post-translational modification and can act in the cell where it is generated. 6. Alternatively, the protein is secreted from the host cell. 7. Antigen protein is degraded by the proteasome in the cytoplasm. The generated antigenic peptide epitopes are transported into the endoplasmic reticulum and loaded onto major histocompatibility complex (MHC) class I molecules (MHC I). 8. The loaded MHC I-peptide epitope complexes are presented on the surface of cells, eventually leading to the induction of antigen-specific CD8^+^ T cell responses after T-cell receptor recognition and appropriate co-stimulation. 9. Exogenous proteins are taken up DCs. 10. They are degraded in endosomes and presented via the MHC II pathway. Moreover, to obtain cognate T-cell help in antigen-presenting cells, the protein should be routed through the MHC II pathway. 11. The generated antigenic peptide epitopes are subsequently loaded onto MHC II molecules. 12. The loaded MHC II-peptide epitope complexes are presented on the surface of cells, leading to the induction of the antigen-specific CD4^+^ T cell responses. Exogenous antigens can also be processed and loaded onto MHC class I molecules via a mechanism known as cross-presentation (not shown in the figure). The figure was created with BioRender.com.

The pharmacodynamic activity of both native and IVT mRNA takes place in the cytosol (Figure 1). However, in contrast to endogenous mRNA, which is transcribed from DNA in the nucleus and enters the cytosol through nuclear export, IVT mRNA enters the cytosol from an extracellular source [18]. Once the IVT mRNA is delivered to the cytosol, its pharmacology is governed by the same complex cellular mechanisms that regulate the stability and translation of endogenous mRNA. The engineered IVT mRNA resembles the endogenous mRNA so closely that the cellular translation machinery is seamlessly utilized to synthesize a protein that may undergo post-translational modifications, eventually resulting in mature protein product(s). In the case of vaccines, this mature protein product(s) represents the antigen(s), which may elicit potent pathogen-specific humoral and cell-mediated immune responses. However, the final intracellular destination is determined by the natural or engineered sequence(s) of the signal peptide or the transmembrane domain [7]. Therefore, mRNA vaccines can be designed for the delivery of the encoded protein(s) to the desired cellular compartment for proper presentation and/or function [19].

The pharmacokinetics of IVT mRNA is determined by the half-life of the mRNA and the resulting mature protein after post-translational modification. The two major factors influencing the bioavailability of exogenous mRNA in the cytosol are (i) rapid RNase-mediated degradation and (ii) lack of passive diffusion across the plasma membrane owing to high molecular weight and electrostatic repulsion between the negative charges of the proteoglycan-coated cell membrane and the negatively-charged mRNA molecules [20]. Naked mRNA is rapidly degraded by extracellular RNases, thus hindering its efficient delivery and efficacy. A wide range of in vitro and in vivo transfection reagents have been shown to protect the mRNA against degradation and facilitate the cellular uptake and endosomal escape of mRNA. Major efforts have been dedicated to improving the enzymatic RNA stability, as discussed further below [3]. Ultimately, the IVT mRNA composed of natural nucleotides is metabolized by inherent physiological mechanisms, hence reducing the risk of metabolite-induced toxicity. Therefore, the delivery issues can be overcome by approaches including encapsulation of mRNA in drug delivery systems consisting of cationic molecules, lipids, polymers, and nanoparticles [21] as well as targeting DCs [22]. In addition, physical transfection methods like electroporation have been shown to enhance the delivery efficiency of large, self-amplifying mRNA in vivo, upon measuring reporter gene expression and immunogenicity of genes encoding HIV envelope proteins [23]. 

## 4. Approaches for Enhancing mRNA Stability 

The development of mRNA-based drugs dates back to 1990 with the successful expression of a number of different proteins upon injecting mRNA encoding these proteins directly into the muscles of mice [24]. This led to (i) the testing of the first mRNA-based vaccine in 1993, which was shown to induce an anti-influenza cytotoxic T-lymphocyte response in mice [25], and (ii) the first vaccination with mRNA-encoding cancer antigens in 1995 [26]. These inceptive demonstrations ratified the potential of mRNA for (i) in situ expression of specific proteins and (ii) for induction of protective antigen-specific cellular and humoral immunity. However, the field was neglected for almost ten years until the potential of in vivo application of mRNA, i.e., induction of specific cytotoxic T lymphocytes and antibodies, was discovered [27]. Advances in the mRNA field were slow due to the labile nature of mRNA, which makes experiments employing unmodified mRNA very challenging unless precautions to handle mRNA are strictly adhered to [28]. Instead, the focus was directed towards DNA-based drugs, since DNA is more stable than RNA. 

In a cell-free system, mRNA can be synthesized by IVT of a DNA template (e.g., a linearized plasmid or a PCR product), which encodes all the structural elements of a functional mRNA. To perform an IVT reaction, all the elements of the natural transcription process are required, i.e., a DNA template, an RNA polymerase, and nucleotide building blocks. During the subsequent purification of the mRNA, the DNA template is often degraded by the addition of DNases, followed by purification by means of other conventional methods for isolating mRNA, e.g., precipitation and chromatography. This process results in highly pure mRNA products ready for use [29,30,31]. Several strategies have been pursued to cope with mRNA’s inherent lack of stability and potential immunogenicity, which are discussed further below. 

### 4.1. Molecular Stabilization

**Figure 2:**
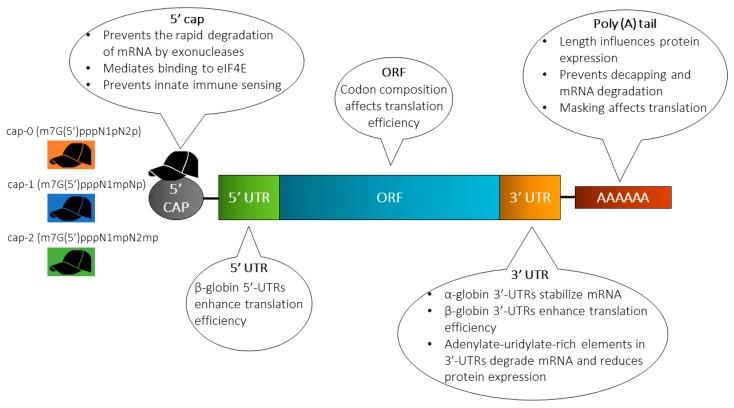
Structure of in vitro transcribed (IVT) mRNA and commonly used modification strategies. The design of IVT mRNA is based on the blueprint of eukaryotic mRNA, and it consists of a 5’ cap, 5’ and 3’ untranslated regions (UTRs), an open reading frame (ORF) encoding antigen(s), and a 3’ poly(A) tail. The IVT mRNA can be modified in one or multiple sites, e.g., by modification of the caps, the UTRs and/or the poly(A) tail, to modulate the duration and kinetic profile of protein expression. eIF4E, eukaryotic translation initiation factor 4E.

Strategies including engineering of sequences and/or structure to enhance mRNA stability (extend the half-life) and translation are often instrumental in increasing the protein expression levels. Techniques employed to achieve this includes elongation of the poly(A) tail, modification of the 5’ cap, engineering of the UTRs and the sequence patterns in the ORF, and/or incorporation of modified nucleotides (Figure 2).

#### 4.1.1. Cap Analog 

A synthetic cap analogue can readily be added to the mRNA because the 5’ end cap is not encoded by the DNA template. Natural eukaryotic mRNA has a 7-methylguanosine (m7G) cap coupled to the mRNA during the transcription process via a 5’-5’-triphosphate bridge (ppp) [32]. The m7GpppN structure at the 5’ end of the mRNA cap serves several functions [33]. First, it protects the mRNA from rapid degradation by exonucleases. Second, it plays an indispensable role during translation because the eukaryotic initiation factor (eIF) 4E recognizes and binds to the cap of the mRNA. It further plays a role in preventing innate immune sensors from recognizing the mRNA [34]. The mRNA may contain one of three distinct caps, i.e., cap-0 [m7G(5’)pppN1pN2p], cap-1 [m7G(5’)pppN1mpNp] and cap-2 [m7G(5’)pppN1mpN2mp], respectively [11]. Capping of IVT mRNA can be performed using two different approaches: The first approach includes the addition of a second step with recombinant vaccinia virus-derived capping enzymes after the transcription, resulting in a cap identical to the most frequent endogenous eukaryotic cap structure i.e., 7-methylguanosine (m^7^G) cap [30]. Alternatively, a synthetic cap analogue may be added during the in vitro transcription reaction, hence capping and in vitro transcription is performed in a single step. This approach is referred to as co-transcriptional capping [35]. A major drawback of this approach is the competition between the cap analog and the GTP nucleotide required for in vitro transcription, which ultimately results in a fraction of uncapped and translationally inactive mRNA [36]. The fraction of uncapped mRNA containing 5’ ppp groups is more immune-stimulating, which can be rectified by treatment with phosphatase to remove the ppp group at the 5’ end of uncapped mRNA [37]. 

Three different classes of m7GpppG cap analogs are used [38]: (i) anti-reverse cap analogs (ARCAs) [39], (ii) 3’-O-Me-m7GpppG [40], and (iii) modified ARCAs [41]. Initial mRNA research was performed using mRNA containing the m7 cap analog (GpppG) [42], and it is currently the most commonly used mRNA cap in clinical trials. Unfortunately, a fraction of the m7GpppG cap analog used during in vitro transcription becomes incorporated in the opposite orientation and is therefore not recognized by the ribosomes, eventually resulting in lower translational activity. To avoid this, the so-called ARCA with only one 3’-OH group instead of two 3’-OH groups (ARCAs; m_2_^7,3’−O^GpppG) has been introduced to prevent incorporation in the opposite orientation [43]. ARCAs juxtaposing the traditional cap analogues have been shown to exhibit more than four times RNA transcription efficiency [44]. In addition, the duration and the levels of protein expression have been found to be enhanced in cells transfected with ARCA-capped IVT RNA [41]. Recently, new types of chemically-modified cap analogues have been introduced, e.g., of phosphorothioate, phosphorothiolate [35], imidiphosphate [45], locked nucleic acid [46], boranophosphate bonds [47] and other types of modifications, which provide the mRNA with resistance to decapping by the mRNA-decapping enzyme 2, eventually resulting in a longer half-life of the mRNA [48]. 

Conventionally, the synthetic 5’-Cap 0-capped RNA is transcribed by performing an in vitro transcription, where more than 80% of the GTP added to the reaction is substituted with a dinucleotide cap analog (i.e., m7G[5’]ppp[5’]G), resulting in initiation of transcription with the cap analog [49]. However, this approach has been shown to exhibit a number of efficiency-related drawbacks, which have been overcome by the introduction of ScriptCap™. ScriptCap™ involves the addition of enzymatically built cap-0 structures onto the RNA transcripts by employing a capping enzyme with 100% reaction efficiency [50]. Native eukaryotic mRNA may contain a cap-1 or cap-2 but never a cap-0 [11]. Therefore, IVT mRNA should contain a cap-1 or a cap-2 in order to be less immune-stimulating [51]. A hallmark of these cap structures is the methylation status of the 2’ position of the 5’ second last and third last nucleoside. Prior to the advent of the novel technology of CleanCap™ introduced by TriLink, synthetic mRNA with a cap-1 could only be prepared by enzymatic capping [52,53]. However, cap-1 or cap-2 can now be incorporated during co-transcriptional capping at a capping efficiency of approximately 94% [52]. Notably, the capping efficiency has been shown to be significantly higher than the efficiency achieved by traditional co-transcriptional capping with cap-0 or ARCA [54].

#### 4.1.2. 5’ and 3’ Untranslated Regions (UTRs) 

The importance of incorporation of 5’- and 3’-UTRs has been noted during in vitro post-transcriptional regulation of gene expression [55]. The numerous roles that UTRs play include (i*)* regulation of mRNA export from the nucleus, (ii) regulation of translation efficiency [56], (iii) orchestration of subcellular localization [57], and (iv) mRNA stability [58]. Introduction of α-globin 3’ end UTRs results in stabilization of mRNA, while the incorporation of beta-globin 5’ end and 3’ end UTRs leads to enhanced translational efficiency [59]. The optimal outcome is achieved by using two β-globin 3’-UTRs aligned in a head-to-tail configuration. α-globin and β-globin UTRs have been incorporated for tweaking the RNA for optimized in vitro transcription followed by mRNA electroporation of autologous T cells [60] and intranodal injection of naked antigen-encoding RNA [61]. Moreover, DCs transfected with antigen-encoding UTR-optimized mRNA have been used in a study involving immunization of cytomegalovirus-seropositive individuals and cancer patients [62]. In some situations, destabilizing the mRNA might be a viable approach to reduce the duration of protein synthesis. This may be accomplished by introducing adenylate-uridylate-rich elements in the 3’-UTRs of the mRNA, eventually resulting in faster mRNA degradation and shortening of the duration of protein expression [63].

#### 4.1.3. Poly(A) Tail

The poly(A) tail plays a significant role in mRNA translation as well as for the enzymatic stability of mRNA. The poly(A) tail binds to several polyadenosyl binding proteins (PABPs) while working synergistically with 5’m7Gcap sequences to regulate translational efficiency [64]. Eukaryotic translation initiation factor eIF4E binds to the 5’m7G cap, which in turn complexes with eIF4G and eIF4A. PABP then interacts with the N-terminus of the eukaryotic translation initiation factor eIF4G, which forms an mRNP (messenger ribonucleoprotein) or a polysome complex [65]. The former depicts the mRNA-protein complex not yet involved in protein synthesis, while the latter is one that is already being translated. An adequately long poly(A) tail is required to circularize the mRNA via binding of PABPs to the poly(A) tail and the cap [55,66]. It has been observed that increasing the poly(A) tail length improves the efficiency of polysome generation and consequently influences the protein expression levels [67]. 

It has been shown that a gradual increase in the poly(A) tail length of IVT mRNA to 120 bases commensurately increases the protein expression level, while an increase in the number of bases beyond 120 does not further enhance protein expression [68]. Poly(A) tails can be added to mRNA by encoding the poly(A) tail in the DNA template, or by extension of the IVT RNA after transcription using recombinant poly(A) polymerase. However, polyadenylation with recombinant poly(A) polymerase results in variable poly(A) tail length, thereby yielding polyadenylated mRNA with varying lengths. Therefore, the preferred approach is the generation of poly(A) tails with well-defined length from the mRNAs transcribed from poly(A) tail-encoding DNA templates [69]. The physical interactions between the 5’ and 3’ ends of mRNA take place between the cap and the poly(A) tail [70]. The poly(A) tail also plays a role in preventing decapping and mRNA degradation because removal or shortening of the poly(A) tail to less than 12 residues results in degradation of the mRNA through cleavage of the 5’ cap structure and 5’ to 3’ exonucleolytic digestion or 3’ to 5’ degradation [71]. 

### 4.2. Formulation Strategies

Despite the promising potential of mRNA-based vaccines, efficient intracellular delivery of mRNA to the cytosol continues to pose a major hurdle, especially for mRNA administered systemically. The large molecular weight (10^5^–10^6^ Da) [21] and high negative charge density of mRNA impair the permeation of mRNA across cellular membranes. It is well known that the absorption of mRNA in the absence of a delivery system is extremely low, and the half-life of mRNA is approximately 7 h [72]. Moreover, mRNA is an inherently unstable molecule, which is highly prone to degradation by 5’ exonucleases, 3’ exonucleases, and endonucleases [73]. Consequently, delivery systems are imperative for intracellular delivery of mRNA to the therapeutic site of action in vitro as well as in vivo [8,74]. Different strategies have been investigated to improve RNA delivery, including improved injection strategies such as microinjections [75], RNA patches [76], gene gun-based administration [77], protamine condensation [78], RNA adjuvants [79], and encapsulation of RNA in nanoparticles consisting of lipids and/or polymers [80]. Generally, IVT mRNA for cytosolic delivery is formulated with a delivery system by mixing with a complexing agent [81], which can protect the mRNA against rapid degradation and facilitate cellular uptake. Although the general dogma in the field is that efficient carriers are needed for substantially enhancing the in vivo transfection of mRNA, naked mRNA have been applied in many in vivo studies. Hence, the following section discusses the delivery of naked mRNA, followed by sections discussing vector-based mRNA delivery [82,83,84,85]. 

#### 4.2.1. Naked RNA

The simplest administration strategy comprises intramuscular (i.m.) injection of naked mRNA, and proof of concept was originally demonstrated by in vivo reporter gene expression in mice [24]. Since then, the effectiveness of naked mRNA has been confirmed upon i.m. [86], subcutaneous (s.c.) [87] or intradermal (i.d.) [88] injections. I.d. and s.c. administration of mRNA has been shown to mediate healing of various skin diseases and to ameliorate wound healing by in situ expression of specific proteins in the skin [75,89]. Adopting this approach circumvents several obstacles otherwise associated with systemic administration of mRNA, e.g., clearance from the bloodstream via the liver, the kidneys, and the spleen [75]. Very efficient translation of the encoded protein has been shown for mRNA administered s.c. [90,91,92]. Interestingly, more efficient translation has at times been measured when compared to mRNA-loaded nanoparticle-based delivery systems [90,93]. Hence, it circumvents the need to employ carriers, eventually contributing to reduced cost and potential risk. Another advantage of administering mRNA-based vaccines via the s.c. route is that both cellular and humoral immune responses are induced [94] because the mRNA is expressed by both skin-resident DCs [95] and non-immune cells [96]. However, the outermost stratum corneum layer of the epidermis serves as a tight barrier to the absorption of topically administered drugs [97]. Heretofore, various approaches have been adopted to overcome this barrier, including physical (e.g., microporation [98], microneedles [99], and jet injection [100,101]), active (e.g., electroporation [102], iontophoresis [103], and sonophoresis [104]), and passive methods (e.g., nanoparticles [105] and liposomes [106]). Electroporation and sonoporation can transiently permeabilize the skin by means of electric pulses and low-frequency ultrasound, respectively, for efficient delivery of genes into the skin [107]. Modified mRNA-encoding vascular endothelial growth factor-A was formulated in citrate-buffered saline without the use of a delivery system and was used for i.d. vaccination of patients with type 2 diabetes [108]. However, in spite of pronounced, sustained, dose-dependent and cargo-specific vasodilation, blood flow increase, oxygen-metabolic upregulation, angiogenesis and neovessel formation in animal models, the vasodilatory and angiogenic activity did not translate into humans [108,109]. Microneedle-based delivery is also an efficient technique where micron-sized needle patch/arrays composed of water-soluble polymeric or sugar excipients are employed, into which the mRNA is incorporated [76]. The patches/arrays provide mechanical strength needed for the needle to permeate the stratum corneum and penetrate into the viable skin layers. Following injection, depending upon the type of microneedles, the patches/arrays degrade/dissolve and the encapsulated drug is released in the interstitial fluid of the skin [75]. Administration of mRNA by using dissolvable microneedles provides an important advantage, i.e., delivery in a solid dosage form circumvents the necessity of dealing with mRNA in a liquid dosage form [110], which therefore eliminates the menace emanating from RNase contamination, along with increasing mRNA stability, and shelf life [76]. Scavenger receptor-mediated endocytosis and micropinocytosis have been shown to be the active uptake mechanisms for naked mRNA in immature DCs [16]. However, naked mRNA displays a short plasma half-life, is prone to ribonuclease degradation, and faces difficulties in entering the cell. Therefore, delivery systems have been propounded to protect the mRNA and shield its negative charge.

#### 4.2.2. Viral Vectors

Delivery of mRNA can be mediated by viral and non-viral vectors. Non-viral vectors can further be categorized into lipid-based delivery systems, polymer-based delivery systems, and lipid-polymer hybrid systems [111]. For viral RNA delivery, there has been a great deal of interest in the engineering of adeno-associated viruses to carry nucleic acid cargoes [112]. Genetically-modified viruses are usually employed for mRNA/gene delivery. The genes of these viruses are partially or fully substituted with model or therapeutic genes. A benefit of RNA viruses is that they are replicated and expressed locally in the cytoplasm. Positive strand RNA viruses are distinguished by a genomic sequence that can be translated directly into proteins of interest by host ribosomes. Notably, alphaviruses (e.g., Sindbis and Semliki Forest virus) [113], picornaviruses [114], and flavivirus [115] (e.g., Kunjin virus) have been employed for mRNA delivery. Various alphavirus vectors can be used to express high levels of exogenous protein in a wide spectrum of hosts [116]. Commonly used approaches include the direct substitution of structural genes with heterologous expression or placing the nonstructural genes downstream of the RNA sub-genomic promoter [117]. However, alphaviruses induce severe cytopathogenic effects, which restrict their application in gene therapy, although different strategies can be employed to surmount this challenge [118]. Some of these strategies include engineering of mutant vectors with mitigated cytotoxicity and temperature-inducibility, and self-inactivating vectors with point mutations in the nsP2 gene (especially at position 726, 259 and 650) [119]. Sendai virus (SeV) (murine parainfluenza virus type 1 or hemagglutinating virus of Japan) that belongs to the Paramyxoviridae family is worth mentioning owing to its popular application as a vector. It is favored for its high but transient gene expression levels, wide host cell specificity, low pathogenicity, and strong immunogenicity [120]. As a vaccine platform, the Venezuelan encephalitis virus is of particular interest [121]. These vaccines encompass the live-attenuated viral vaccine TC-83 and a formalin-inactivated variety of it is referred to as C-84, which boosts the efficacy and increases the duration of immunity upon administering the TC-83 vaccine via different administration routes, intranasal being the most widely used [122]. However, using viral vectors embodies crucial drawbacks associated with genome integration, and possible host rejection (immunogenicity and cytotoxicity) among others [123], hence provoking the need for non-viral vectors for mRNA delivery [10]. 

#### 4.2.3. Polymer-Based Vectors

Diethylaminoethyl (DEAE) dextran was the first polymer to be tested as a delivery reagent for IVT mRNA [124]. Later, it was shown that lipid-mediated mRNA transfection is 100 to 1000 times more efficient than DEAE-dextran [125]. This discovery stalled the progress of polymeric carriers and paved the way for lipid-based transfection reagents for nucleic acids, including mRNA. A comprehensive study compared the polymers poly-beta-amino-esters (PBAE) and polyethylenimine (PEI) with commercial transfection reagent Lipofectamine™ 2000 and 1,2-dioleoyl-3-trimethylammonium propane (DOTAP)/ 1,2-dioleoyl-sn-glycero-3-phosphoethanolamine (DOPE) for functional, antigen-specific T-cell responses after mRNA delivery [126]. All carriers were complexed with mRNA encoding the HIV-1 antigen *gag*. Gag-specific, IFN-γ secreting T cells were measured in the spleen and lymph nodes of mice immunized with gag mRNA complexed with cationic lipids but not in mice immunized with naked and polymer-complexed mRNA. PEI and its derivatives are among the most commonly employed cationic polymers [127]. They are water-soluble, display a high density of positive charge associated with the amino groups, and are proven mRNA carriers for in vitro transfection [128]. However, PEI displays toxicity issues owing to the high molecular weight (>25 kDa), which may arise from the adsorption of anionic serum proteins onto the polyplex surface between cationic polymers and anionic serum plasma proteins. However, the resultant increase in size is only transient as the proteins adsorbed on the surface of polyplexes prevent particle–particle aggregation in the long run [129]. Various efforts have been made to mitigate these challenges. The first proof of concept for safe and efficacious mRNA vaccine transfection employing cationic polymer was obtained by intranasal administration of 2 kDa PEI conjugated to cyclodextrin. Cyclodextrin conjugated to PEI enabled delocalization of the charge density on the polyamine backbone, hence reducing cytotoxicity and at the same time maintaining protonatable groups, resulting in improved transfection [130]. Polymeric nanoparticles composed of biodegradable polymers, e.g., poly(lactic-*co*-glycolic acid) (PLGA), are well suited for incorporation of hydrophobic and positively-charged molecules. They provide good colloidal stability, low toxicity, and the possibility of sustained release. However, due to the anionic nature of PLGA at physiological pH [131], the mRNA encapsulation efficiency is very low. Polymer-based carriers exhibit considerable potential for gene therapy owing to the substantial transfection efficiency and tolerable toxicity [132]. A series of multifunctional block copolymers, i.e., dimethylaminoethyl methacrylate (DEAMA), poly(ethylene glycol) methacrylate, and DEAEMA-co-n-butyl methacrylate, demonstrated a transfection efficiency of 77% and 50% in RAW 264.7 macrophages and DC2.4 dendritic cells, respectively, thereby exhibiting potential as a carrier for mRNA-based intracellular vaccine delivery [133]. While different types of polymers and copolymers have been tested, the correlation between the structure of polymers and their biological response, e.g., transfection and toxicity, was found to be poor and thus, design of various polymer-based delivery systems relies on empirical, rather than rational approaches [134]. Despite the advantages mentioned above, polymer-based delivery systems are not as clinically advanced as lipid-based delivery systems owing to their polydispersity and challenges pertaining to metabolism of large molecular weight polymers [21].

#### 4.2.4. Lipid-Based Vectors

Vectors based on lipids or lipid-like compounds (lipidoids) represent the most commonly used non-viral gene carriers [21]. Various synthetic and naturally-derived lipids have been employed to form liposomes (complexes of liposomes and nucleic acids are referred to as lipoplexes) or lipid nanoparticles (LNPs), both of which have been reported to efficiently deliver mRNA-based vaccines (Table 2). LNPs are often formulated by using cationic lipids displaying tertiary or quaternary amines to encapsulate the polyanionic mRNA. Cationic lipids spontaneously encapsulate negatively-charged mRNA, mediated by a combination of attractive electrostatic interactions with RNA and hydrophobic interactions, and thus have been used alone or in combination for lipofection of mRNA. The first reported use of LNPs as delivery system for mRNA came in 2015, with the delivery system consisting of ionizable cationic lipid/phosphatidylcholine/cholesterol/PEG-lipid in the ratio of (50:10:38.5:1.5 mol/mol) [86]. Examples of cationic lipids include e.g., 1,2-di-O-octadecenyl-3-trimethylammonium propane (DOTMA) [135], DOTAP [136], and zwitterionic DOPE [137,138]. They are structurally denoted by a cationic headgroup, a hydrophobic tail group, and a linking group in between [139]. However, cationic lipids have been observed to exhibit pro-inflammatory reactions and undesirable side effects [140]. Therefore, neutral lipids are also incorporated into cationic liposomes to decrease toxicity and attain high transfection levels in vivo [106]. The mechanism of LNP-mediated delivery of mRNA is not fully understood, but LNPs are suggested to be internalized by endocytosis and are attached electrostatically and fused with the cell membrane via inverted non-bilayer lipid phases [21]. 

Liposomes are closed membrane structures, which are formed by self-assembly when phospholipids are dispersed in aqueous systems [141]. They consist of at least one phospholipid bilayer, which mimics the cell membrane structure enclosing an aqueous core [8]. DOTAP/DOPE at a 1:1 molar ratio has been reported as an effective transfection agent for mRNA encoding the HIV-1 antigen Gag, which successfully induced an antigen-specific immune response in vivo in mice [126]. Additionally, 3β-[N-(N’,N’-dimethylaminoethane) carbamoyl](DC)-Cholesterol)/DOPE-based liposomes in a [1:2] ratio achieved high encapsulation efficiency of enhanced green fluorescent protein (eGFP) mRNA, along with high eGFP expression in vitro [106]. Furthermore, an additional multi-component LNP displayed a tumor-suppressant effect when loaded with herpes simplex virus I (HSV I) thymidine kinase encoding mRNA. The LNPs were composed of DOTAP/Cholesterol [1:1] liposomes along with 1,2-distearoyl-phosphatidylethanolamine (DSPE)-polyethylene glycol (PEG) and DSPE-PEG-anisamide (AA) [142]. The principle behind their effectiveness may be summarized as a combination of its electrostatic interactions attributable to opposite charges and hydrophobic interactions with mRNA. Additionally, the endosomal escape capabilities and self-assembling properties resulting in uniform layers enclosing polymeric cores also contribute to the wide application of cationic lipids [143]. However, in vivo studies are more challenging due to the fast elimination of cationic lipids by the mononuclear phagocytic system [144]. Cationic lipids consisting of only one quaternary ammonium headgroup pose safety issues such as toxicity and immunogenicity in vitro [145] and in vivo [146]. For instance, cationic liposomes when administered via the intravenous route may induce hepatotoxicity [147] and can trigger a strong IFN-γ response in mice resulting in inflammation [148,149]. Furthermore, positively-charged lipids, e.g., DOTAP and DOTMA, can be neutralized by anionic serum proteins, leading to toxicity and reduced efficacy [150]. Moreover, challenges like unrestricted protein binding, colloidal instability, and drug leakage may arise [151]. 

Alternatively, new gene delivery vectors containing ionizable lipids [152] and lipid-like materials termed lipidoids [153] have been introduced to overcome challenges posed by conventional cationic lipids while retaining their advantageous transfection properties. Ionizable lipids for mRNA transfection are positively-charged at low pH (which aids in mRNA complexation when it is carried out in acidic buffer) but are neutral at physiological pH (for reduced toxicity post-injection) [154]. Unlike conventional cationic lipids, these lipidoids display a series of secondary and tertiary amines allowing for more efficient interactions with mRNA without remarkably increasing the overall charge of the delivery system [155]. Encapsulation of mRNA in nanoparticles serves to physically protect nucleic acids from degradation and depending on the specific chemistry can aid in cellular uptake and endosomal escape [156]. The combination of the ionizable lipid C12-200, cholesterol, DOPE and C14-PEG2000 at a 3.5:4.65:1.6:0.25 molar ratio, respectively, encapsulating erythropoietin mRNA (EPO-mRNA) displayed high efficacy in vivo when injected into mice, measured as the cellular expression of EPO [157]. The emphasis on the nanoparticle platform for mRNA delivery is in part due to the application of established DNA and siRNA delivery systems. 

#### 4.2.5. Lipid-Polymer Hybrid Nanoparticles

Lipid-polymer hybrid nanoparticles (LPNs) have been demonstrated previously to exhibit effective functional delivery of siRNA in vitro [158] and therapeutic delivery of siRNA in vitro and in vivo [159]. This hybrid delivery system has also shown promising results for delivery of mRNA, with the mRNA being encapsulated in a hybrid nanoparticle composed of the lipid-like material N1, N3, N5-tris(2-aminoethyl)benzene-1,3,5-tricarboxamide (TT) in TT3:DOPE:Cholesterol:DMG-PEG2000 (1,2-dimyristoyl-sn-glycerol, methoxypolyethylene glycol) with a polymeric PLGA core [160]. In addition, optimized LPNs consisting of the degradable polymer PBAE, formulated with PEG-Lipid C14-2000, showed successful delivery of mRNA to the lungs [161]. This, along with reported co-delivery of siRNA and mRNA with lipidoid polymer hybrid nanoparticles [162], shows that LPNs are an emerging nucleic acid delivery system. The hybrid formulation is thermodynamically favorable, with respect to hydrophobic, van der Waal, and electrostatic interactions [80]. Several lipids and polymers have been investigated to formulate stable nucleic acid lipid particles using this delivery system. Most common polymers employed are PLGA, polycaprolactone, polylactic acid, or their combinations, whereas the lipids used include DOTAP, 1,2-dilauroyl-sn-glycero-3-phosphocholine, 1,2-distearoyl-sn-glycero-3-phosphocholine, lecithin, DSPE, and PEG, among others. Structurally, based on small-angle X-ray scattering and cryogenic transmission electron microscopy, these nanoparticles are suggested to entail a polymeric matrix core with lamellar lipid structures with the nucleic acid localized in the core and in the corona [163].

#### 4.2.6. Peptide-Based Vectors

Peptide-based systems for mRNA delivery are gaining momentum due to the versatility peptides can offer. Peptide-based delivery systems, both alone and in combination with other materials such as polymers, have been reported in the literature. In a study concerning ovarian cancer therapy, the commercially available cell-penetrating peptide (CPP) PepFect14 was complexed with eGFP mRNA via attractive electrostatic interactions [164]. This nanoparticulate formulation was more efficient in transfecting eGFP mRNA into cells associated with ovarian cancer than commercially available lipofectamine MessengerMAX. Similarly, the CPP RALA has been used to effectively deliver both eGFP and OVA mRNA and has been demonstrated to outperform the cationic lipid DOTAP and the fusogenic lipid DOPE [165]. However, current limitations include targeted cell delivery [164] and short circulation half-life due to the low stability in serum-containing medium [166]. 

Recently, a novel polymer-peptide hybrid mRNA delivery nanoplatform was introduced [167] combining both polymer (PLA) based micelles and a cationic fusogenic peptide (RALA) to achieve appropriate degradability, mRNA stability, and endosomolytic properties for translation. It was reported to protect eGFP as well as FLuc mRNA against serum nuclease degradation and achieve DC transfection. Indeed, peptide-based vectors and hybrids are promising and interesting additions to the various existing non-viral carriers for the delivery of mRNA.

### 4.3. Cell-Specific mRNA Delivery

Cell-specific delivery of mRNA would be beneficial for the development of mRNA-based therapeutics. This can enhance the delivery of mRNA molecules to the targeted cells and hence reduce the required mRNA dose, as well as reducing potential off-target effects. It has been reported that lymphoid organs can be targeted by adjusting the net charge of the formulation [177]. This is based on the principle of APCs being in the vicinity of T cells in these organs, thus providing optimal conditions for efficient priming and amplification of T-cell responses. Site-specific delivery of mRNA-loaded nanoparticles via active targeting has been shown to result in induction of strong effector and memory T-cell responses, and mediation of potent IFN-α-dependent rejection of progressive tumors, as observed with RNAs. In another study, cell-specific delivery of FLuc and IL-10 mRNA to leukocytes (Ly6c+) was achieved by coating the formulated mRNA-containing LNPs with anti-L6c+ monoclonal antibodies [178]. Alternatively, DCs and macrophages express receptors with the ability to present antigens, e.g., C-type lectin receptors [179], which recognize sugar groups such as mannose- and fucose-terminated glycans [180] and mediate the endocytosis of mannose-modified nanoparticles. This has been exploited for transfection of GFP mRNA into DCs by self-assembly of mannose-cholesterol conjugates with varying PEG units as linkers [181].

## 5. Conclusions and Future Perspectives

The field of mRNA-based therapeutics spans from protein replacement therapy and gene editing to vaccination. With the dozens of mRNA-based vaccine candidates currently in pre-clinical and clinical phases of development, it is evident that the mRNA-based vaccine technology is a promising tool for the development of novel therapeutic and prophylactic vaccines against infectious diseases and cancer. However, the multifarious obstacles associated with mRNA’s extremely large size, charge, intrinsic instability, and high susceptibility to enzymatic degradation hamper the translation of mRNA-based therapeutics from the bench to the bedside. Therefore, the wider application of mRNA-based therapeutics is still limited by the need for improved vectors or drug delivery systems. Advanced delivery systems can be applied to overcome the poor stability, cell targeting, and translational efficiency of naked mRNA. However, many clinically tested mRNA vaccine candidates are formulated without any delivery system, which suggests a need for further improvement of delivery systems for mRNA vaccines. Presently, lipoplexes and lipid-based nanoparticles are mostly used for delivering mRNA. Additionally, polymers and lipid-polymer hybrid nanoparticles offer great promise in terms of safety, stability, high transfection efficiency, and low price. Continued advancement in mRNA formulation and delivery using different nanomaterials can improve the wider use of mRNA for the treatment and prevention of infectious diseases and cancers.

## Figures and Tables

**Table 1:** Examples of ongoing clinical trials of mRNA-based vaccines.

mRNA	Mechanism of Action	Disease/Condition	Administration Route	Study Phase	Sponsor/Collaborator	National Clinical Trial Identifier
**Therapeutic** **mRNA**						
W_ova1 vaccine	Induction of an anti-tumor immune response	Ovarian cancer	Intravenous	Phase I	University Medical Center Groningen/BioNTech	NCT04163094
CT7, MAGE-A3, and WT1 mRNA-electroporated Langerhans cells (LCs)	Electroporation of dendritic cells with antigen mRNA	Multiple Myeloma	Subcutaneous	Phase I	Memorial Sloan Kettering Cancer Center	NCT01995708
Personalized Cellular mRNA	Immunization with DCs pulsed with mRNA encoded tumor antigens	Brain cancer/Neoplasm Metastases	Not specified	Phase I	Guangdong 999 Brain Hospital/Beijing, Tricision, Trinomab, Jinan University Guangzhou	NCT02808416
Personalized mRNA	Immunization with DCs pulsed with personalized mRNA	Glioblastoma	Not specified	Phase I	Guangdong 999 Brain Hospital/Beijing Tricision, Trinomab, Jinan University Guangzhou	NCT02808364
MiHA mRNA	Immunization with DCs loaded with MiHA mRNA	Hematological malignancies	Intravenous	Phase I Phase II	Radboud University/ZonMw: The Netherlands Organization for Health Research and Development Dutch Cancer Society	NCT02528682
WT1-mRNA	Immunization with DCs electroporated with WT1-mRNA	Acute myeloid leukemia	Not Specified	Phase II	Zwi Berneman/Kom Op Tegen Kanker stichting tegen kanker Research Foundation - Flanders (FWO: Fonds Wetenschappelijk Onderzoek)	NCT01686334
Human CMV pp65-LAMP mRNA	Immunization with DCs pulsed with CMV pp65-LAMP mRNA	Glioblastoma	Intradermal	Phase II	Gary Archer Ph.D./Duke University	NCT03927222
Personalized mRNA	Personalized mRNA tumor vaccine encoding neoantigen	Advanced esophageal squamous carcinoma, gastric adenocarcinoma, pancreatic adenocarcinoma, colorectal adenocarcinoma	Subcutaneous	Enrolling	Changhai Hospital/Stemirna Therapeutics	NCT03468244
mRNA[BI 1361849 (formerly CV9202)]	Not specified	Metastatic non-small cell lung cancer	Not specified	Phase I Phase II	Ludwig Institute for Cancer Research/Cancer Research Institute, New York City, Boehringer Ingelheim MedImmune, CureVac, PharmaJet	NCT03164772
mRNA-5671/V941	Not specified	Neoplasms, carcinoma, non-small-cell lung, pancreatic neoplasms, colorectal neoplasms	Intramuscular	Phase I	Merck Sharp & Dohme	NCT03948763
mRNA-4157	Immunostimulants	Solid tumors	Not specified	Phase I	Moderna/Merck Sharp & Dohme	NCT03313778
mRNA-4157	Immunotherapy with the personalized cancer vaccine	Cutaneous melanoma	Not specified	Phase II	Moderna/Merck Sharp & Dohme	NCT03897881
Personalized mRNA	Encoding neoantigen	Esophageal cancer Non-small cell lung cancer	Subcutaneous	Enrolling	Stemirna Therapeutics/The First Affiliated Hospital of Zhengzhou University	NCT03908671
mRNA-3704	Alpha-galactosidase stimulants; Methylmalonyl CoA mutase stimulants; Protein synthesis stimulants	Methylmalonic acidemia, metabolism, inborn errors	Intravenous	Phase I Phase II	Moderna	NCT03810690
mRNA-2416	OX40 ligand modulators	Relapsed/Refractory solid tumor malignancies or lymphoma	Intratumoral	Phase I	Moderna	NCT03323398
mRNA-2752	IL36G protein stimulants; Interleukin 23 stimulants; OX40 ligand modulators	Relapsed/Refractory solid tumor malignancies or lymphoma	Intratumoral	Phase I	Moderna/AstraZeneca	NCT03739931
**Prophylactic mRNA**						
mRNA-1647, mRNA-1443	Not specified	Cytomegalovirus	Not specified	Phase I	Moderna	NCT03382405
mRNA-1893	Not specified	Zika virus	Not specified	Phase I	Moderna/Biomedical Advanced Research and Development Authority	NCT04064905
mRNA-1653	A combined human metapneumovirus and human parainfluenza virus type 3 vaccine	Human metapneumovirus and Human Parainfluenzavirus	Not specified	Phase I	Moderna.	NCT03392389 NCT04144348
mRNA-1944	Encoding for an anti-Chikungunya virus monoclonal antibody	Chikungunya virus	Parenteral	Phase I	Moderna	NCT03829384
mRNA-1653	Immunostimulants	Metapneumovirus and Parainfluenza virus	Parenteral	Phase I	Moderna	NCT03392389
CV7202	Immunostimulants	Rabies	Intramuscular	Phase I	CureVac	NCT03713086

**Table 2:** Examples of nanoparticulate drug delivery systems for mRNA delivery.

Drug Delivery Systems	Composition	RNA	Disease/Condition	References
**Polymers**	Poly(glycoamidoamine)	Erythropoietin (EPO) mRNA	Anemia and myelodysplasia	[168]
	Polyethyleneimine	HIV-1 gag mRNA	HIV	[169]
	Poly(β-amino ester) (PBAE)	eGFP mRNA	N/A	[170]
	Triblock copolymer (comprising DMAEMA, PEGMA, DEAEMA and BMA)	eGFP and ovalbumin	N/A	[171]
	DEAE-Dextran	Luciferase-encoding mRNA	N/A	[172]
**Lipids**	DOTAP/DOPE	HxB-2 HIV-1 Gag antigen mRNA	HIV	[126]
	DOPE/DC-Cholesterol [2:1]	eGFP mRNA	N/A	[106]
	DOTAP/Cholesterol [1:1] liposome with DSPE-PEG and DSPE-PEG-AA	HSV I Thymidine kinase mRNA	Cancer	[142]
	C12-200:Cholesterol: DOPE:C14-PEG2000	EPO mRNA	N/A	[157]
	A18	Ovalbumin mRNA	Melanoma	[173]
	cKK-E12	HER2 antibody mRNA	Cancer	[174]
	(6Z,9Z,28Z,31Z)-heptatriaconta-6,9,28,31-tetraen-19-yl 4-(dimethylamino)butanoate (MC3), DSPC, cholesterol, and 1,2-dimyristoyl-rac-glycerol, methoxypolyethylene glycol (PEG2000-DMG)	Human erythropoietin		[175]
	DOTAP/DOPE [1:1]	HIV-1 antigen Gag mRNA	HIV	[126]
	3β-[N-(N’,N’-dimethylaminoethane) carbamoyl](DC-Cholesterol)/DOPE (1:2)	eGFP mRNA	N/A	[106]
**Lipid polymer hybrid NPs**	TT3:DOPE:Cholesterol:DMG-PEG2000 with PLGA core	Firefly luciferase (FLuc) mRNA and eGFP mRNA	N/A	[160]
	PBAE:C14-PEG2000	FLuc mRNA	N/A	[161]
	PBAE:EDOPC/DOPE/DSPE-PEG	Ovalbumin mRNA	N/A	[176]
	PBAE: DOPC, DOTAP, and DSPE-PEG	eGFP mRNA	N/A	[105]
**Peptides and peptide-polymer hybrids**	PepFect14	eGFP mRNA	Ovarian cancer	[164]
	RALA	eGFP mRNA OVA mRNA	N/A	[165]
	RALA-PLA	eGFPmRNA FLuc mRNA	N/A	[167]

BMA: butyl methacrylate; DEAE: diethylaminoethyl; DEAEMA: diethylaminoethyl Methacrylate; DMAEMA: dimethylaminoethyl acrylate; DOPE: dioleoylphosphatidylethanolamine; DOTAP: dioleoyl-3-trimethylammonium propane; DSPC: dipalmitoylphosphatidylcholine; DSPE-PEG: 1,2-distearoyl-sn-glycero-3-phosphoethanolamine-N-[amino(polyethylene glycol); DSPE-PEG-AA: DSPE-PEG-anisamide; eGFP: enhanced green fluorescent protein; HER2: human epidermal growth factor receptor 2; HIV: human immunodeficiency virus; HSV: herpes simplex virus; N/A: not applicable; PEGMA: poly(ethylene glycol) methacrylate; PLGA: poly(lactic-co-glycolic acid); TT: N1,N3,N5-tris(2-aminoethyl)benzene-1,3,5-tricarboxamide; PLA: polylactic acid.
